# Interferons: Reprogramming the Metabolic Network against Viral Infection

**DOI:** 10.3390/v10010036

**Published:** 2018-01-13

**Authors:** Kavita Raniga, Chen Liang

**Affiliations:** 1 Lady Davis Institute for Medical Research, Jewish General Hospital, Montreal, QC H3T 1E2, Canada; kavita.raniga@mail.mcgill.ca; 2 Department of Microbiology & Immunology, McGill University, Montreal, QC H3A 2B4, Canada; 3 Department of Medicine, McGill University, Montreal, QC H3A 2B4, Canada

**Keywords:** viruses, metabolism, interferons, ISGs, 25HC, IDO1, SAT1, SAMHD1

## Abstract

Viruses exploit the host and induce drastic metabolic changes to ensure an optimal environment for replication and the production of viral progenies. In response, the host has developed diverse countermeasures to sense and limit these alterations to combat viral infection. One such host mechanism is through interferon signaling. Interferons are cytokines that enhances the transcription of hundreds of interferon-stimulated genes (ISGs) whose products are key players in the innate immune response to viral infection. In addition to their direct targeting of viral components, interferons and ISGs exert profound effects on cellular metabolism. Recent studies have started to illuminate on the specific role of interferon in rewiring cellular metabolism to activate immune cells and limit viral infection. This review reflects on our current understanding of the complex networking that occurs between the virus and host at the interface of cellular metabolism, with a focus on the ISGs in particular, cholesterol-25-hydroxylase (CH25H), spermidine/spermine acetyltransferase 1 (SAT1), indoleamine-2,3-dioxygenase (IDO1) and sterile alpha motif and histidine/aspartic acid domain-containing protein 1 (SAMHD1), which were recently discovered to modulate specific metabolic events and consequently deter viral infection.

## 1. Introduction

Cellular metabolism is a fundamental and complex biological phenomenon. It consists of two opposite and entwined processes, catabolism that breaks down macromolecules to produce energy in the form of adenosine triphosphate (ATP) and fuel all cellular reactions and events; and anabolism that delivers nutrients such as carbohydrates, amino acids and fatty acids for macromolecular synthesis [1]. The fine balance of catabolic and anabolic pathways is critical for meeting diverse biological demands, thus allowing organisms to respond actively to dynamic environments. 

Abnormal metabolic states are the hallmark of neurological disorders, diabetes, obesity and cancer. It is increasingly evident that viral infections not only rewire the metabolism of the host cell to the advantage of viral propagation but, can leave “metabolic footprints” causing metabolic disease [2]. In turn, to respond and contain viral infections, the hosts and, in particular, their immune cells, need to selectively mobilize and deploy their metabolic resources in order to mount effective innate and adaptive immune responses. 

While not the focus of this review, it is well received that immune cells need to adopt distinct metabolic profiles to achieve their activation, differentiation and effector function [3,4]. Several articles have detailed the significant metabolic signatures of immune cells, such as macrophages, dendritic cells and T-cells, upon activation [5,6,7]. Early studies indicated a change in metabolism towards aerobic glycolysis for lipopolysaccharide (LPS)-activated macrophages, which is required to produce pro-inflammatory cytokines such as IL-1β [5]. This metabolic signature is also apparent in T cells, where elevated levels of aerobic glycolysis are maintained in effector T cell subsets in response to cytokines. In particular, the pro-inflammatory lymphocyte Th17 heavily depends on glycolysis for differentiation where, if glycolysis is blocked, Th17 differentiation and survival is impaired and an anti-inflammatory phenotype is favored [8]. Importantly, this shift to aerobic glycolysis allows immune cells to adapt, survive and acquire their effector functions when under metabolically limited conditions during infection or hypoxia. 

In this review, we focus our discussion on the metabolic interplay between viruses and the innate immune response that occurs at the very early stage of viral infection of the host. We illuminate what is known about how various viruses modulate cellular metabolism by distinct mechanisms and how cells, in response to viral infections, produce interferons to alter specific metabolic pathways as one effective strategy to control viral replication.

## 2. Viral Rewiring of Central Carbon Metabolism

Viruses are completely dependent on their host’s cellular metabolism to fuel the production of viral progenies. The effects of viruses on cellular metabolism were already described in studies from the 1950s, when it was observed that addition of glucose into the minimal media significantly increased the yield of polioviruses from the infected HeLa cells [9]. However, a detailed understanding of this subject was considerably accelerated with the application of mass spectrometry to measure many metabolites concurrently as well as the possibility of tracking carbon flux using isotope labelled carbon sources including glucose and glutamine [10,11]. Extensive metabolic studies have now been performed on a variety of viruses, including dengue virus [12], human herpesvirus type 8 (HHV8) [13], hepatitis C virus (HCV) [14], vaccinia virus [15], human immunodeficiency virus type 1 (HIV-1) [16], influenza A virus (IAV) [17], human cytomegalovirus (HCMV) [18], and herpes simplex virus-1 (HSV-1) [18]. The results have revealed three core metabolic pathways: glycolysis, fatty acid synthesis and glutaminolysis that are commonly altered by almost all viruses using distinct mechanisms to facilitate discrete steps of viral propagation as well as sustain the latently infected cells. 

**Figure 1:**
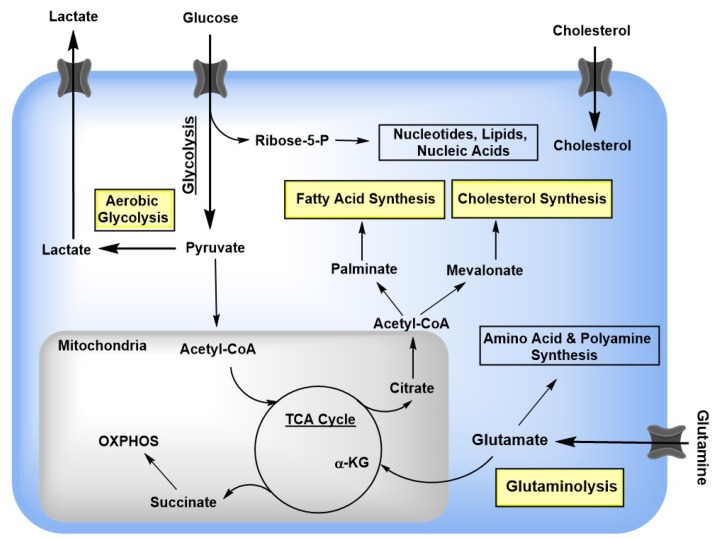
Host Cell Metabolism. Glucose is taken up by specific transporters (GLUT family), where it is converted to pyruvate in the cytoplasm, generating two ATP molecules (glycolysis). In the presence of oxygen, pyruvate is transported into the mitochondria and oxidized into acetyl coenzyme A (acetyl-CoA), which enters the tricarboxylic acid (TCA) cycle. Intermediates of the TCA cycle feed off for fatty acid and cholesterol synthesis. Viruses are known to alter these key cellular metabolic pathways (highlighted in yellow). Further details are outlined in the text.

Glycolysis is a multiple step process that converts glucose to pyruvate concurrent with the production of 2ATPs and the reduction of two nicotinamide adenine dinucleotide (NAD) to NADH (Figure 1). In normal non-proliferating cells that are exposed to oxygen, pyruvate enters mitochondria where it is oxidized into acetyl coenzyme A (CoA). CoA then feeds the tricarboxylic (TCA) cycle (also known as citric acid or Krebs cycle) and drives the electron transport chain to generate ATP from adenosine diphosphate, called oxidative phosphorylation [19]. Once completely metabolized into carbon dioxide through the TCA cycle, one glucose can generate 36 ATP equivalents. In the lack of oxygen, in addition to entering the TCA cycle, pyruvate is also reduced to lactate. This type of glycolysis can also occur in the presence of sufficient oxygen in tumor cells, a process called aerobic glycolysis or the Warburg effect which was first described by Otto Warburg [20]. It is now known that viral infection can cause a shift to aerobic glycolysis to generate a rapid source of ATPs and also, using the abundant metabolic intermediates in the presence of reduced TCA cycle, to accelerate the synthesis of nucleotides, amino acids and fatty acids that are needed for assembly of a large number of viral progenies in a relative short time span. Glutamine is the second carbon source. In a process called glutaminolysis, glutamine is metabolized to alpha ketoglutarate (αKG) that can enter the TCA cycle to generate ATPs (Figure 1). Some viruses can use both carbon sources, while others, such as vaccinia virus, prefer to use glutamine [15]. In addition, fatty acids also serve as an important energy source, which can be oxidized in mitochondria to produce CoA that drives the TCA cycle. 

Viruses appear to have evolved a plethora of mechanisms to modulate the flux of exogenous carbon sources (including glucose, glutamine, fatty acids and amino acids) through the complex and yet well-coordinated metabolic highways, with the sole outcome to generate energy and biomass molecules that together ensure massive production of virus particles. Detailed accounts of this important subject are provided in recent reviews [21,22,23]. 

In addition to the emerging commonality of viral alteration of cellular metabolism, it is also worth noting that different viruses have evolved distinct strategies to modulate cellular metabolism to support viral propagation. Even between closely related viruses, such as two herpesviruses HCMV and HSV-1, their manipulations of cellular metabolic pathways are elegantly tailored to meet their different metabolic needs. Although both HCMV and HSV-1 alter glycolysis, in contrast to HCMV that increases glucose uptake and causes aerobic glycolysis, HSV-1 diverts glucose from the TCA cycle to drive nucleotide synthesis, which supports rapid viral DNA replication to keep pace with its much faster viral replication cycle (24 h) compared to that of HCMV (96 h) [18]. The metabolomics analysis of viral alteration of cellular metabolism has painted a rich picture of the complex and fascinating metabolic interactions between viruses and their hosts. A deeper understanding of these metabolic exchanges will need the identification of the underlying viral mechanisms, including which viral gene products target which key factors in specific metabolic pathways. 

In addition, it is worthwhile elucidating which steps of viral propagation benefit from the virally induced changes in a specific metabolic pathway. For example, although Kaposi’s sarcoma-associated herpesvirus (KSHV) alters glycolysis, glutaminolysis and fatty acid synthesis for efficient viral production, glycolysis appears to support the transcription of early genes; glutaminolysis is needed for translation of early genes, whereas fatty acid synthesis promotes the late steps of production of extracellular infectious virions [24]. These details are expected to guide the development of drugs that block specific metabolic processes that are key to the propagation of a specific pathogenic virus yet impose marginal or manageable side effects on the hosts. While viruses modulate the metabolism of host cells, the hosts have also developed strategies to redirect cellular metabolic traffics to inhibit virus production. One such prominent host mechanism is interferon. 

## 3. Diverse Antiviral Mechanisms by Interferons and Their Stimulated Genes

Type I Interferons (IFNs) were discovered over half a century ago because of their strong antiviral activity against influenza virus [25]. Subsequent studies have revealed the intricate signalling pathways that are triggered by IFNs, leading to the expression of hundreds of genes, collectively called interferon-stimulated genes (ISGs), many of which have been shown to inhibit a variety of viruses through diverse mechanisms [26]. Some ISGs directly target and impair specific steps of viral replication. For example, IFITM (interferon inducible transmembrane) proteins deter viral entry; the Mx (myxovirus resistance) proteins recognize viral capsid/nucleocapsid and impede viral genome replication; tetherin (also called BST-2 and CD137) physically links viral progenies to the surface of viral producer cells and thus blocks virus release and dissemination [26]. Furthermore, a group of ISGs inhibit the translation of viral RNA, thus halt viral replication. These ISGs include PKR (protein kinase R), 2′-5′-oligoadenylate synthetase (OAS)/RNAseL, IFIT1, ZAP (zinc finger antiviral protein), SLFN11 and others, which was discussed in detail in a recent review [27]. Our knowledge of the antiviral functions of ISGs was greatly expanded with several recent investigations that tested hundreds of ISGs for their inhibition of a wide range of viruses [28,29,30,31,32]. In addition to direct inhibition of viruses, some ISGs are professional viral sensors, such as RIG-I, PKR, IFI16, cGAS and others [33,34]. Beyond all these functions and mechanisms, an emerging new antiviral strategy employed by ISGs is to modulate cellular metabolic pathways, including the synthesis of cholesterol, polyamines and tryptophan.

## 4. 25-HC Inhibits a Variety of Viruses by Modulating Sterol Synthesis

Cholesterol is an abundant lipid on the cell membrane, responsible for membrane packing and fluidity. Replication of many viruses, including viral entry and viral budding, are affected by the distribution of cholesterol in cellular membranes and, are thus hindered when cholesterol biosynthesis is inhibited [35]. Transcription factors namely, sterol regulatory element-binding proteins (SREBPs), tightly regulate the biosynthesis of cholesterol and fatty acids. With regards to viral infection, SREBPs are strongly linked to HCV pathogenesis whereby the virus upregulates SREBP activity by stimulating PI3K/AKT signaling [36]. Consequently, an antiviral mechanism employed by the immune response involves transcriptional downregulation of SREBP2 to attenuate viral replication [37].

**Figure 2:**
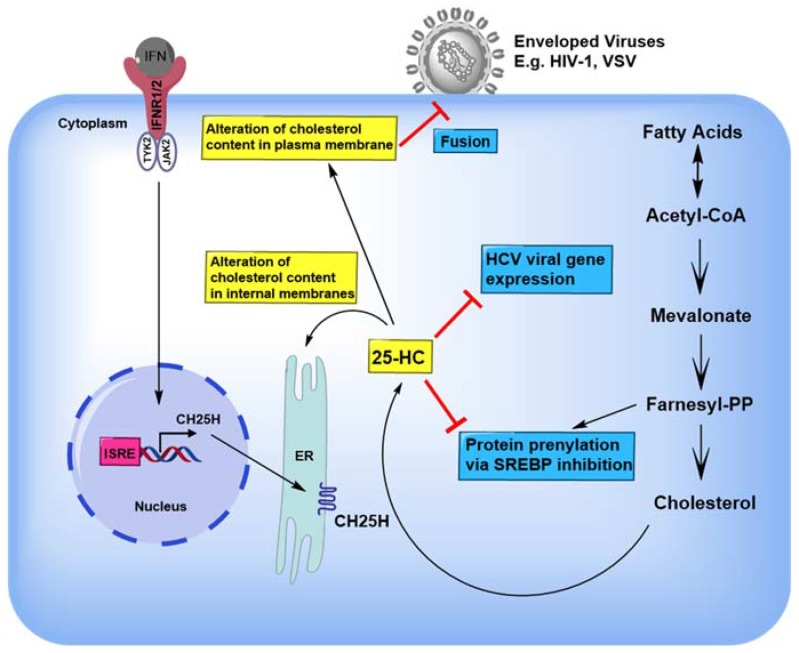
An overview of the antiviral activities of 25-HC. Interferon signaling leads to the expression of CH25H (cholesterol 25-hydroxylase) which catalyzes the formation of 25 hydroxycholesterol (25-HC) from cholesterol. Multiple studies have elucidated into the antiviral effects of 25-HC including, altering lipid membrane content to restrict viral fusion and entry, altering the distribution of cholesterol in internal membranes which hinders viral replication and, antagonizing endogenous protein prenylation (attachment of an isoprenoid e.g., farnesyl) thereby restricting viral replication and assembly. Yellow boxes indicate membrane alterations induced by 25-HC. Blue boxes indicate the effect of 25-HC on different viruses. T bar in red indicates inhibition. ER, endoplasmic reticulum.

In the quest for antiviral cellular factors targeting sterol pathways came the discovery of the antiviral ISG Cholesterol-25-hydroxylase (CH25H) that encodes an enzyme which converts cholesterol to the soluble oxysterol, 25-hydroxycholesterol (25-HC) [38]. CH25H is potently expressed in macrophages and dendritic cells in response to activation of toll-like receptors (TLRs), through the production of type I and II IFNs (Figure 2) [38]. CH25H-induced 25-HC has a well-defined role in the regulation of sterol biosynthesis, reducing cholesterol accumulation, thus executing its antiviral cellular functions. 25-HC may do so also by repressing SREBP2 activation and/or by enhancing the expression of microRNA miR-185 that regulates hepatic lipid homeostasis, which remains to be fully elucidated [39,40].

Cell culture assays have shown that 25-HC inhibits multiple enveloped viruses, including HIV-1 [38,41], HCV (42), vesicular stomatitis virus (VSV) [42], Ebola virus [42], SFTS virus (SFTSV) [43], West Nile Virus (WNV) [44], Dengue virus [44], Zika virus (ZIKV) [44] and, interestingly, Pseudorabies virus (PRV) the etiological agent of Aujeszky’s disease in pigs [45]. Despite studies reporting 25-HC to be inactive against non-enveloped viruses [38], other studies have shown marked 25-HC antiviral activity against polio virus [46], human papillomavirus-16 (HPV-16), human rotavirus (HRoV), and human rhinovirus (HRhV) [47]. More recent studies into the antiviral role of 25-HC has extended beyond human viral pathogens, to investigating porcine viruses and even the role of 25-HC in non-mammalian species such as zebrafish [48,49]. Interestingly, phylogenetic analysis identified CH25Hb as the homolog of mammalian CH25H in zebrafish however, its expression was not modulated by type 1 IFN. Nevertheless, studies showed that overexpression of CH25Hb had a protective effect on zebrafish larvae following Spring Viremia of Carp virus (SVCV) infection [49]. In support of the results of many cell culture based assays, 25-HC has been shown to suppress HIV-1 infection in humanized mice [38]. More recently, it was reported that 25-HC reduces Zika virus (ZIKV) viremia and improves survival in mice and rhesus monkeys [44], which attests to the potential therapeutic value of 25-HC as an antiviral agent.

25-HC exerts it antiviral function by several mechanisms (Figure 2). The first mechanism involves the alteration of the cellular membrane composition, which impacts multiple stages of the replication of different viruses, ranging from viral entry to viral genome replication to viral gene expression at the cytoplasmic membrane structures. A study conducted by Liu and colleagues demonstrated that 25-HC induces cellular membrane modifications resulting in impaired viral entry during virus-cell fusion [38]. 25-HC specifically alters the cellular membrane by reducing syncytia formation, to inhibit HIV-1 entry without affecting transcription, translation or budding of HIV-1 [38]. Likewise, 25-HC blocks ZIKV entry into the cell in a dose-dependent manner [44]. The inhibitory role of 25-HC in HCV infection has also been extensively studied. Electron microscopy analysis revealed 25-HC inhibited the formation of a membranous web (HCV replication site) on intracellular membranes [42]. 

The second mechanism for the antiviral action of 25-HC works by post-translational modifications (PTMs). Glycosylation is the most common PTM, and occurs to many viral envelope proteins [50]. Shrivastava-Ranjan and colleagues demonstrated that 25-HC inhibits Lassa virus (LASV), an arenavirus, through causing aberrant GP1 glycosylation [51]. The LASV genome consists of two RNA segments (large (L) and small (S)); the small segment encodes a glycoprotein precursor (GPC), which is subsequently cleaved into GP1 and GP2. GP1 and GP2 mediate viral attachment to host cell receptors and the fusion of viral and endosomal membranes, respectively. N-linked glycosylation of GP1/GP2 is critical for its transportation to sites of viral budding to carry out their functions [52]. Shrivastava-Ranjan and colleagues showed 25-HC impaired LASV production and infectivity through inhibiting GP1 glycosylation, leading to the incorporation of aberrantly glycosylated GP1 into LASV particles [51]. The precise mechanism by which 25-HC induces these changes has yet to be elucidated. 

To add to the multiple antiviral mechanisms by 25-HC, one study delved into the relationship between immune mechanisms and the antiviral effects of 25-HC. Cagno and colleagues reported that induction of 25-HC promotes the production of IL-6 following HSV-1 virus infection [53]. For non-enveloped viruses such as Polio virus, 25-HC exerts its antiviral activity through interacting with oxysterol-binding protein (OSBP) [54]. OSBPs are sensors, coordinators and/or transporters of cholesterol from the ER to the Golgi [55]. Experiments showed that 25-HC interferes directly with the transport of cholesterol from the ER to the Golgi, resulting in a reduction in cholesterol accumulation at sites of viral replication [56]. Similarly, 25-HC inhibited the replication of Rhinoviruses by binding to OSBP with high affinity, locking it in an “inactive state” [57].

It is evident from these studies that the sterol pathway, including 25-HC, plays a pivotal role in viral infection and is conserved across species. Nevertheless, while the host attempts to downregulate lipid uptake in this manner, reports have suggested that TLRs and IFNs can conversely increase lipid uptake from the environment leading to foam cell formation [58,59,60]. To address the role of IFN in this context, York and colleagues found that TLRs and type 1 IFNs decreased de novo cholesterol biosynthesis yet, increased cholesterol uptake [61]. Surprisingly, this metabolic shift could itself activate type 1 IFN signaling revealing a novel IFN-mediated mechanism [61]. Clearly, further studies are warranted to determine the precise molecular interactions of the immune response with 25-HC as well as SREBP activation to develop promising drug candidates. 

## 5. IFNs Deplete Polyamines as a Strategy to Check Viral Infection

**Figure 3:**
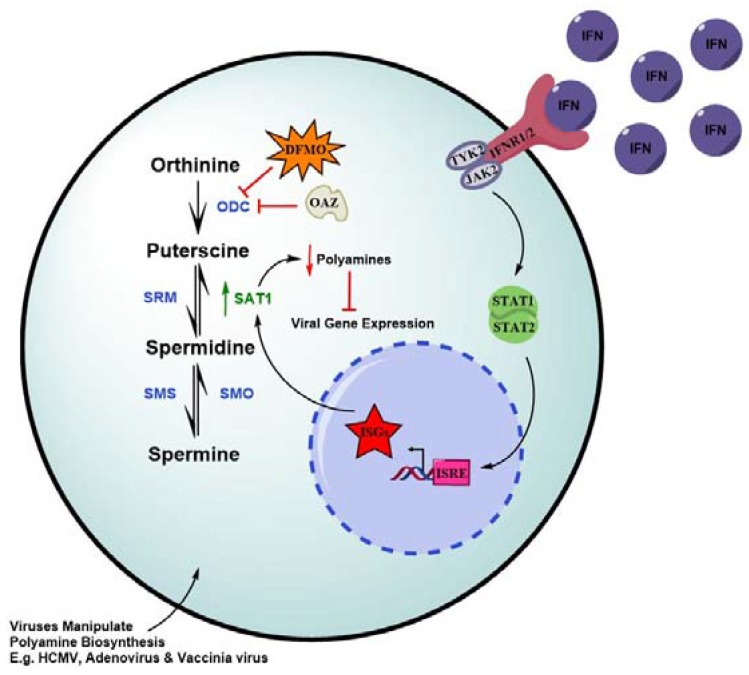
Polyamine Biosynthesis. Upregulation of SAT1 by Type 1 IFNs results in polyamine depletion (red arrow). Enzymes are shown between each reaction (ODC, orthinine decarboxylase; SRM, spermidine synthase; SMS, spermine synthase; SMO, spermine oxidase; SAT1, Spermidine/spermine *N*(1)-acetyltransferase). Ornithine decarboxylase antizyme (OAZ), the first rate-limiting step in polyamine synthesis, binds and inhibits ODC. Difluoromethylornithine (DFMO), currently in clinical trials for cancer treatment, is an irreversible inhibitor of ornithine decarboxylase. T bar indicates inhibition. Blue dotted circle, nucleus. Consult text for more detail.

Polyamines have been long known to promote the replication of many DNA viruses and RNA viruses yet, only until recent was it reported that IFNs modulate levels of polyamines and consequently suppress the life cycles of polyamine-dependent viruses [62,63]. Polyamines are ubiquitous, small, positively charged biogenic molecules essential for promoting cell growth and differentiation [64], mediating apoptosis [65] and protecting cells against oxidative stress [66]. The three mammalian polyamines, derived from ornithine, are putrescine, spermidine and spermine. There are several rate-limiting enzymes which determine intracellular levels of polyamines. These include ornithine decarboxylase (ODC) which generates putrescine, and spermidine/spermine acetyltransferase 1 (SAT1) which is involved in the regulation of polyamine concentration via catabolizing spermine back to spermidine and putrescine (Figure 3) [67]. Interestingly, SAT1 expression responds to the stimulation by type I interferon and therefore, is a bona fide ISG [68,69]. 

Owing to their intense positive charge, polyamines bind to DNA and RNA in cells, and regulate the function of nucleic acids through altering their structure and conformation [70]. It is estimated that 80% of polyamines are associated with cellular RNA [71]. Therefore, it is not surprising to find polyamines in the virions of many DNA viruses including HSV-1 [72], HCMV [73], vaccinia virus [74], where polyamines neutralize the negative charges of viral DNA genome, assist packaging viral DNA into virus particles, and also enhance viral DNA synthesis in the new round of viral replication. Recent studies further revealed that a large number of RNA viruses are also dependent on polyamines for replication, these include Semliki forest virus, Chikungunya virus (CHIKV), ZIKV, Middle East respiratory syndrome coronavirus(MERS-CoV) , enterovirus 71, Ebola virus, Marburg virus, HIV-1, and many others reviewed in [63]. 

The underlying mechanisms include promoting the synthesis of viral RNA likely as a result of tight binding of polyamines to viral RNA, and accelerating viral protein translation. This latter mechanism of action stems from the fact that spermidine is a substrate of deoxyhypusine synthase that is a key enzyme accounting for the hypusination of translation initiation factor eIF5A [75,76]. Recently published, polyamines and hypusinated eIF5A have been shown to be critical for Ebola virus gene expression and replication [77]. The important role of polyamines in viral replication was further supported by the results of studies showing that depletion of polyamines using difluoromethylornithine (DFMO), a suicide inhibitor of ODC1 which is a rate-limiting enzyme of polyamine synthesis, significantly suppresses sindbis virus (SINV) infection of Drosophila melanogaster and zebrafish Danio rerio [68]. 

The exploitation of polyamines by viruses is also evident in light of the viral mechanisms that manipulate the biosynthesis of polyamines. HCMV, adenovirus, and vaccinia virus enhance the activity of ODC1, which catalyses the conversion of ornithine to putrescine in the polyamine synthesis pathway [63]. The paramecium bursaria chlorella virus 1 (PBCV-1) even encodes the whole polyamine biosynthesis pathway, including ornithine/arginine decarboxylase, homospermidine synthase, agmatine iminohydrolase (AIH) and N-carbamoylputrescine amidohydrolase (CPA), which suggests the importance of putrescine and polyamines in the life cycle of PBCV-1 [78]. In the meantime, HCV appears to inhibit the synthesis of spermine and spermidine from putrescine, the implications of which in viral replication needs further investigation [79].

The dependence of so many viruses on polyamines to replicate has, over evolution, inevitably endowed the host an opportunity to form mechanisms to modulate polyamine metabolism so that viral replication can be suppressed. Indeed, recent studies showed that interferon-β treatment leads to depletion of spermine and spermidine concurrent with increased putrescine as a result of SAT1 induction. Knockout of SAT1 restored putrescine level in the presence of interferon-β and significantly recovered the infection of CHIKV, demonstrating depletion of polyamines through induction of SAT1 represents one mechanism of IFN antiviral function [68]. 

As we investigate into the regulation of the polyamine pathway by interferon, viral proteins and/or the changes in the cellular environment, we begin to uncover multiple pathways of cross-protection. Currently, targeting polyamine metabolism has been directed towards a therapeutic/preventative treatment for cancer. For example, DFMO, an irreversible inhibitor of ODC, has recently attracted interest for the treatment of neuroblastoma [80,81]. With regards to treating viral infections, one recent study in mice showed that polyamine depletion limited CHIKV replication [82]. DFMO is a stable and well-tolerated drug with mild side-effects in humans and can be administered via several routes including, orally meaning, it could hold promise in treating viral infections [83].

## 6. IFN-Mediated Depletion of Tryptophan through IDO1 Induction

**Figure 4:**
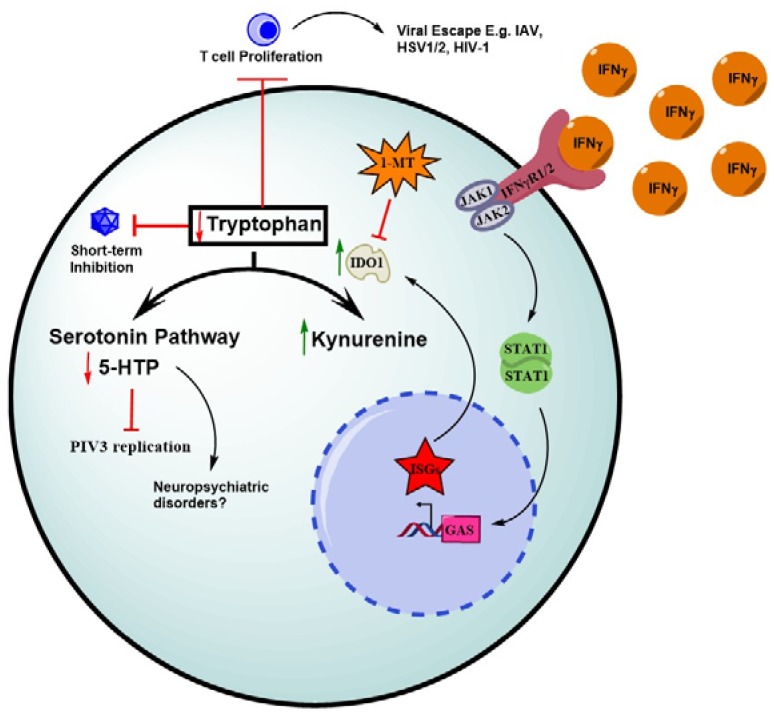
IDO1-mediated Tryptophan Depletion. Tryptophan is metabolized by the serotonin and kynurenine pathway. Iindole-2,3-dioxygenase 1 (IDO1) converts tryptophan into kynurenine. IFN-induced IDO1 gene expression decreases tryptophan availability for viral replication as well as reducing melatonin/serotonin synthesis and T cell proliferation. 1-methyl-d-tryptophan (I-MT) inhibits IDO1 and may have applications as a facilitator to enhance the immune response. Red arrows, downregulation; green arrows, upregulation. T bar, inhibition.

L-Tryptophan (L-Trp), discovered in the early 1900s, is one of the nine essential amino acids and is critical in several metabolic pathways, primarily protein, kynurenine, and serotonin synthesis [84]. While the least abundant out of all the 20 amino acids, L-Trp and its catabolites have significant roles in disease tolerance and immunosuppression [85]. Importantly, the kynurenine pathway accounts for greater than 90% of tryptophan catabolism [86]. The synthesis of kynurenine (Kyn) derivatives from tryptophan is initiated through the enzymatic activity of indoleamine-2,3-dioxygenase (IDO1) and tryptophan-2,3-dioxygenase (TDO2) (Figure 4) [87]. The ISG IDO1 is highly expressed across many cell types, whereas TDO2 is mainly expressed in hepatocytes and has a lower affinity for tryptophan [88]. IDO1 is thus significantly important for tryptophan homoeostasis and, not surprisingly, is positioned at the intricate interface of host–virus interactions.

First, host cells increase IDO1 expression in response to viral infections. This is because the promoter region of IDO1 gene contains two interferon-stimulated response elements (ISREs) and three IFNγ-activated sites that respond to interferons that are often produced to control viral infections [89]. While type-1 IFNs can also induce IDO1 expression, IFNγ remains the most potent inducer of IDO1 expression [90]. As viruses are metabolically inert, they are highly susceptible to IDO1-mediated L-Trp deprivation. The growth of a range of viruses is inhibited by IDO1, such as HIV (30), IAV [91], HSV-2 [92], HBV [93], HCV [94], vaccinia virus [95] and parainfluenza virus (PIV3) [96].

In a comprehensive screen for antiretroviral ISGs, IDO1 was identified for its strong anti-HIV-2 activity [30]. The study subsequently confirmed that production of both HIV-1 and HIV-2 was substantially curtailed by IDO1 as a result of severe suppression of viral protein synthesis. Since supplementation of L-tryptophan or inhibition of IDO1 with its competitive inhibitor 1-methyl-L-Tryptophan (1-MT) fully rescued HIV production in IDO1-expressing cells, it is IDO1-created depletion of L-Tryptophan that has blocked viral protein synthesis. The significant role of IDO1 in IFNγ-mediated strong inhibition of HIV-1 production was demonstrated by the 10-fold rescue of HIV-1 yield in A549 or TZM-bl cells that were treated with either L-Tryptophan or the IDO-inhibitor 1-methyl-d-tryptophan(1-MT) [30]. 

In an effort of screening of ISGs inhibiting PIV3, Rabbani and colleagues confirmed that the anti-PIV3 activity of IDO was due to tryptophan depletion, by adding exogenous tryptophan or inhibiting the activity of IDO by 1-MT to counteract the antiviral effect of IDO in vitro [96]. Results showed adding tryptophan concentrations of 50 μg/mL or higher, or increasing the amount of 1-MT rescued PIV3 replication. Further, Rabbani and colleagues investigated into the parallel serotonin-biosynthetic pathway which is initiated by Tryptophan hydroxylase (TPH) with the first product being 5-hydroxytryptophan (5-HTP) [96]. They showed that addition of 5-HTP in IDO-expressing cells rescued PIV3 replication. In addition, using an inhibitor of Trp hydroxylase (THP), the group showed that 5-HTP is a proviral factor [96]. 

As long-term induction of IDO expression leads to immunosuppression, several viruses use this as an advantage to promote their own replication [97]. Gaelings and colleagues studied the regulation of the kynurenine pathway during IAV infection, as it was previously reported that IAV induced the production of kynurenine [98,99]. Primary macrophages and mouse lungs infected with IAV showed IFN-mediated upregulation of IDO1 however, this IDO1 activity was attenuated by the IAV NS1 protein through suppressing interferon production [98]. In addition to IAV, other viruses such as influenza B virus, HSV1/2 also lead to the upregulation of the kynurenine pathway in immune cells [98]. 

Another important aspect to consider is the role of the serotonin pathway during infection. A clear example comes from models of lymphocytic choriomeningitis virus (LCMV)-induced hepatitis which leads to aggregation and activation of platelets [100,101]. In mice, these platelets secreted serotonin in the liver which resulted in hepatic sinusoid microcirculation failure and enhanced T-cell cytotoxicity [102]. These studies provide a unique perspective in the role of serotonin during infection and permit further investigation. 

Because of the immunosuppressive outcome due to long-term IDO1 expression, IDO1 plays a counterintuitive role during chronic HIV-1 infection. L-Trp depletion results in a shift to an immunotolerogenic microenvironment, whereby there is an increase in the production of immunomodulatory kynurenine derivatives and T cell responses are suppressed, which may accelerate disease progression [103,104]. A recent study using an SIV-infected pigtailed macaque model also reported evaluation of kynurenine pathway metabolites and enzymes in lymphoid tissues [105]. Interestingly, their results showed no significant reduction in tryptophan levels in the spleen or brain despite, a substantial depletion in the plasma and cerebrospinal fluid (CSF). Based on these findings, Drewes and colleagues proposed that tryptophan depletion is not a primary contributor to T-cell impairment during HIV/SIV infection [105], which challenges the previous reports regarding the role of the kynurenine pathway during acute and chronic viral infections and warrants further research into this important subject.

IFN-mediated IDO1 upregulation upon various acute and chronic viral infections has been demonstrated by many research groups. However, it is a double-edged sword. While exerting antiviral effects against numerous viruses, long-term consequences of induction could contribute to disease progression, as seen with the promotion of an immunosuppressive environment during chronic viral infections [106]. Nonetheless, the immunosuppressive nature of IDO could be used as an advantage. Promising results showed that the 1-MT reduced the size and growth of tumors in mice preimmunized with a tumor antigen [107]. With regards to infection, early studies demonstrated that mice treated with 1-MT had higher CD4+ T cell and effector memory CD8+ T-cell counts [108]. Recent studies have shown that suppressing IDO in combination with a natural killer cell stimulator and an influenza vaccine boots protective immunity against influenza [109,110]. Furthermore, mice treated with 1-MT and infected with IAV had an elevated CD4+, CD8+ and effector memory T-cell response in the lungs compared to control groups [111]. The same group also showed an increase in PRR and pro-inflammatory cytokines upon IAV infection [112]. These results suggest the use of 1-MT as a facilitator to enhance the immune response against infection in combination with adjuvants and vaccination. Nevertheless, one should be cautious, as multiple pathways are affected upon tryptophan depletion and exact mechanisms remain unknown. 

## 7. SAMHD1 Restricts Viral DNA Synthesis by Depleting Cellular dNTP Pool

Propagation of large amounts of viral progenies in a short time demands the synthesis of large quantities of viral genomes, which requires the adequate supply of nucleotide substrates by the cells, and also becomes a weakness of viral replication that cells are able to take advantage of. One prominent example of cellular antiviral strategy along this line is the sterile alpha motif and histidine-aspartate domain containing protein 1 (SAMHD1) that inhibits the DNA synthesis of not only HIV-1 but a broad range of retroviruses and DNA viruses and including, HIV-2, murine leukaemia virus (MLV), human T cell leukemia virus type 1, vaccinia virus, HSV-1 and HBV [113,114,115,116,117,118]. 

Prior to its discovery as an antiviral factor against viruses, SAMHD1 gene mutations were found to associate with the autoimmune neurological condition, the Aicardi–Goutières syndrome (AGS) [119]. AGS affects new-born infants and mimics a congenital viral infection with elevated interferonα levels in cerebrospinal fluid [120]. 

The *SAMHD1* gene was first identified in human dendritic cells as an ortholog of the mouse gene, Mg11 induced by interferon-γ upon viral infection [121]. Subsequent studies have also shown type 1, type II IFNs, and toll-like agonists upregulate SAMHD1 expression in multiple cell types, including murine macrophages [122], PBMCs (6), monocytes [123], and microglia [124]. Interestingly, SAMHD1 mRNA levels were upregulated in astrocytes although protein levels remained unchanged [124]. In the meantime, studies also reported that monocyte-derived dendritic cells, primary CD4+ T cells and monocyte-derived macrophages do not upregulate SAMHD1 expression upon interferon treatment [125,126]. This inconsistency in SAMHD1 responsiveness to interferon stimulation is partially because SAMHD1 is not a conventional ISG in a sense that its promoter does not bear the classic IFN-responding DNA motifs, but rather its expression is negatively controlled by a couple of microRNAs (miRNAs), including miR181a and miR30a, that bind to the 3′ untranslated region (UTR) of SAMHD1 mRNA [123]. Riess and colleagues expressed constructs containing SAMHD1 3′ UTR in HEK293T cells or U037 cells and demonstrated that the 3′ UTR is required for interferon-induced SAMHD1 expression. This group also showed that miRNAs negatively regulated SAMHD1 expression namely, miR-181a, miR-30a and miR-155. To further support this, induction with IFN downregulated miR-181a and miR-30a 24 h post IFN stimulation [123]. These findings suggest that SAMHD1 expression is increased by interferons through decreasing miR30a and miR181a. 

SAMHD1, a dNTP hydrolase, regulates intracellular dNTP levels by catalysing the conversion of deoxynucleoside triphosphates to deoxynucleoside and inorganic triphosphate [127]. Thus, the antiviral activity of SAMHD1 is through depleting the pool of dNTPs necessary for viral DNA synthesis and viral replication [128]. Certain studies have also suggested that SAMHD1 has RNase activity, which was disputed by the results from other groups [129,130,131,132]. Studies in SAMHD1 knockout mice show a significant increase in intracellular dNTP concentrations and in reverse transcription of retroviruses whose reverse transcriptase has low affinity to dNTP [133,134]. 

As a key regulator of cellular dNTP pool, the activity of SAMHD1 has been subject to modulation by both cellular and viral mechanisms. First, the expression level of SAMHD1 is cell cycle dependent [135,136]. To warrant sufficient dNTP levels to support cellular DNA duplication at the S phase, the cyclin-dependent kinases CDK2 and CDK6, part of the mechanisms for controlling cell cycle progression, phosphorylate T592 of SAMHD1, which, by yet to be defined molecular mechanisms, prevents SAMHD1 from depleting dNTPs [136].

An alternative cellular mechanism involves a membrane protein called CD81 that binds to SAMHD1 and interacts with SAMHD1 and directs SAMHD1 toward proteasomal degradation [137]. On the side of viruses, HIV-2 and SIV encode an accessory protein called Vpx that recognizes SAMHD1 and recruits it to the E3 ubiquitin ligase complex for ubiquitination and subsequent proteasomal degradation [113,114]. It is intriguing that HIV-1, a much more successful pathogen than HIV-2 in causing a global HIV pandemic lasting for decades already, has not bothered to form a mechanism to counter SAMHD1. Among the possible explanations, one is that the high levels of SAMHD1 in resting CD4+ T cells impairs the synthesis of HIV-1 DNA [138]. These aborted viral DNA fragments trigger pyropoptosis through activating IFI16, the DNA sensor, which leads to depletion of CD4 cells [139]. Additionally, SAMHD1 inhibits HIV-1 infection of DCs, which delays the sensing of HIV-1 infection and the onset of immune responses, consequently leaving the virus a longer time window to establish local infection, the foot hold for later systemic infection [125]. 

## 8. Targeting Metabolic Pathways for Viral Therapy: A Novel Therapeutic Approach

Viral infections remain a significant challenge to human health, ranging from seasonal outbreaks of influenza affecting 20% of the global population to recent outbreaks of Zika virus, Dengue virus, Ebola virus and middle east respiratory syndrome coronavirus (MERS-CoV) [140]. Due to the appearance of these new emerging and re-emerging viruses and, given the fact that many antivirals are highly specific to viral targets, there is a clear need to develop new drugs and find novel therapeutic targets against viral infections, specifically, broad-spectrum (i.e., pan-genotypic or pan-family) antiviral drugs that are accessible and affordable for low income countries. 

Viruses are metabolic engineers which dramatically affect host cell metabolism and the virus life cycle, providing a novel avenue for therapeutic intervention. A prime example is the IAV. IAVs evolve continuously and can cause pneumonia, encephalitis, myocarditis or even multiple organ failure and death. Antiviral drugs targeting viral factors are available, however, studies have shown a rise in viral resistance with neuraminidase inhibitors [141,142,143,144]. Smallwood and colleagues recently identified metabolic changes in IAV-infected human lung epithelial cells [145]. These changes, observed in pediatric patients infected with respiratory viral infections, include increases in glucose and glutamine uptake, fatty acid metabolism, TCA cycle and oxidative phosphorylation compared to non-infected patients. Moreover, through conducting targeted drug screens with 80 metabolic drugs, Smallwood and colleagues identified the drug BEZ235 which reduced viral titers in vitro and in vivo, significantly increased survival in mice and reversed influenza-induced metabolic changes [145]. BEZ235, an inhibitor of the PI3K-AKT- mTOR metabolic pathway, is currently a drug candidate under clinical trials for the treatment of cancer [146,147]. It is thought BEZ235 exerts its antiviral effect by limiting the availability of glucose and glutamine to IAV [145]. Much work has yet to be conducted on understanding the mechanisms behind the antiviral effect of BEZ235 and the long-term consequences. Given the similarity in the metabolic profiles between cancer cells and virus infected cells, it may not come as a surprise that more drug candidates used in the treatment of cancer will be shown effective in controlling pathogenic viruses.

Another example came from the detailed analysis of metabolic changes in primary hepatocytes upon HCV infection [148]. In agreement with others, Levy and colleagues demonstrated that HCV-infected primary hepatocytes underwent a Warburg-like shift in glycolysis while downregulating oxidative phosphorylation. Importantly, concomitant transcriptional regulatory analysis of glycolysis identified an enrichment of two nuclear receptors, HNF4α and HNF1α. HNF4α is key in regulating lipid homeostasis, gluconeogenesis as well as cell proliferation and apoptosis [149,150,151]. In support of this finding, the addition of HNF4α antagonists, Medica16 and BI6015, or an siRNA against HNF4α reduced glycolysis and significantly decreased lactate production. In addition, upon addition of HNF4α antagonists, a three- to fourfold decrease in viral RNA levels was observed [148]. Again, this study supports the concept that reversing the metabolic changes caused by viruses, using chemical compounds, may serve as an attractive therapeutic strategy to control viral infection.

As fatty acid and cholesterol synthesis are enhanced upon viral infection to support the generation of new viral progenies, it comes to no surprise that 5′-adenosine monophosphate-activated protein kinase (AMPK) is also activated. AMPK is an intracellular sensor, that maintains metabolic homeostasis through regulating glucose and lipid metabolism [152,153]. Activated AMPK inhibits fatty acid and cholesterol synthesis while upregulating fatty acid oxidation [153]. Dysregulation of AMPK is implicated in metabolic syndrome-related diseases. Studies have shown activated AMPK can restrict a number of RNA viruses including, Rift Valley fever virus (RVFV), Bunyavirus, SINV and VSV [154]. A recent paper investigated AMPK activation in response to KSHV primary infection [155]. Results showed that inhibition of AMPK significantly enhances KSHV lytic replication. Given the importance of AMPK in cellular metabolism multiple drugs have been developed to treat metabolic disorders.

One such drug is 5-aminoimidazole-4-carboxamide ribonucleotide (AICAR), an AMP analog, which can stimulate AMPK activity. Studies conducted using AICAR to treat ischemia and type II diabetes have shown promise [156,157]. With regards to viral infection, the group observed inhibition of KSHV lytic genes upon AICAR treatment corresponding to the suppression of lytic replication. Furthermore, metformin, the first-line drug for managing type II diabetes also decreased viral yields by 60% and also inhibited the activation of KSHV lytic genes [155]. Overall, these results suggest AMPK could be used as a therapeutic target to treat viral infections. However, AMPK, an evolutionary conserved kinase has pivotal roles in multiple signaling pathways and prolonged activation could have unwanted consequences. Further, virus-specific effect from AMPK also warrants careful testing of AMPK inhibitors on individual viruses. 

We need to be cautious that viruses may still escape from treatments that target cellular factors and pathways. A recently published paper aimed to address the issue of antiviral resistance if compounds targeting polyamine synthesis were used as a potential treatment [158]. Prolonged virus passaging in cells treated with the drug difluoromethylornithine (DFMO) resulted in the production of CHIKV mutants resistant to polyamine depletion, in vitro. Sequencing analysis of the viral population identified three distinct mutations: G230R and V326M in non-structural protein 1 (nsP1) and a change of the opal stop codon into arginine. Interestingly, individually expressed mutations conferred no resistance to polyamine depletion however, in vitro and in vivo studies revealed that combinations of mutations enhanced CHIKV replication and fitness and allowed the virus to resist DMFO treatment [158]. Through immunoprecipitation studies, the two mutations in nsp1 enhanced membrane binding and promoted genome methylation, while the mutation of the opal sop codon enhanced downstream translation. These results highlight the strategy that CHIKV has developed to survive polyamine depletion through selecting for mutations that elevate viral fitness. While targeting host cell metabolism provides a unique opportunity to restrict viral propagation, one needs to consider the potential negative effects on immune cell function. In particular, both virus infected cells and activated immune cells tend to adopt aerobic glycolysis, which generates sufficient amounts of energy and macromolecules, in a relatively short time period, to support rapid virus replication and cell proliferation. Therefore, selective targeting cell metabolic steps will be the key for an effective and successful antiviral therapy that inhibits viral propagation, but not immune cell functions.

## 9. Concluding Remarks

As viruses have evolved mechanisms to target the host metabolic network to ensure survival, simultaneously, the hosts have accordingly selected distinct antiviral mechanisms to counteract these changes. The innate immune response plays a pivotal role in responding to these viral changes in effort to warrant cell survival and limit viral propagation. A robust IFN response leads to the priming of the adaptive immune response through modulating the metabolic pathways in innate immune cells (macrophages and DCs) as well as, the induction of ISGs and restriction factors which interfere with cellular metabolism. Several of these ISGs, CH25H, SAT-1, IDO1 and SAMHD1, exert antiviral activity against multiple viruses including DENV, ZIKV, HCV, HIV-1 and HSV by targeting different metabolic pathways. Currently, a limited number of ISGs have been reported to modulate cellular metabolism. Future studies, including gene expression and pathway analysis of the Interferome database, could potentially lead to discovery of more ISGs linked to metabolism. 

The role of IFNs and ISGs in immunometabolism and regulating carbon metabolism has opened novel avenues for viral therapy. The development of mass-spectrometry and gene expression screening has led to a deeper understanding of virus-host metabolic interactions. However, much remains to be discovered regarding the detailed molecular mechanisms of these metabolic interactions and the potential the clinical applications. It is important to note that targeting metabolic pathways for therapeutic intervention may lead to fitness costs to the host. However, if these potential side effects are short-term and manageable, as seen in many medications that target cellular components, interfering with certain pathways may be overall beneficial and may lead to the production of broad spectrum antivirals.

## References

[1] DeBerardinis R.J., Thompson C.B. (2012). Cellular metabolism and disease: What do metabolic outliers teach us?. Cell.

[2] Slama L., Le Camus C., Serfaty L., Pialoux G., Capeau J., Gharakhanian S. (2009). Metabolic disorders and chronic viral disease: The case of HIV and HCV. Diabetes Metab..

[3] Bantug G.R., Galluzzi L., Kroemer G., Hess C. (2018). The spectrum of T cell metabolism in health and disease. Nat. Rev. Immunol..

[4] Phan A.T., Goldrath A.W., Glass C.K. (2017). Metabolic and Epigenetic Coordination of T Cell and Macrophage Immunity. Immunity.

[5] Tannahill G.M., Curtis A.M., Adamik J., Palsson-McDermott E.M., McGettrick A.F., Goel G., Frezza C., Bernard N.J., Kelly B., Foley N.H. (2013). Succinate is an inflammatory signal that induces IL-1beta through HIF-1alpha. Nature.

[6] Buitendijk M., Eszterhas S.K., Howell A.L. (2014). Toll-like receptor agonists are potent inhibitors of human immunodeficiency virus-type 1 replication in peripheral blood mononuclear cells. AIDS Res. Hum. Retrovir..

[7] Gerriets V.A., Rathmell J.C. (2012). Metabolic pathways in T cell fate and function. Trends Immunol..

[8] Buck M.D., O’Sullivan D., Pearce E.L. (2015). T cell metabolism drives immunity. J. Exp. Med..

[9] Eagle H., Habel K. (1956). The nutritional requirements for the propagation of poliomyelitis virus by the hela cell. J. Exp. Med..

[10] Munger J., Bennett B.D., Parikh A., Feng X.J., McArdle J., Rabitz H.A., Shenk T., Rabinowitz J.D. (2008). Systems-level metabolic flux profiling identifies fatty acid synthesis as a target for antiviral therapy. Nat. Biotechnol..

[11] Munger J. (2006). Dynamics of the Cellular Metabolome during Human Cytomegalovirus Infection. PLoS Pathog..

[12] Fontaine K.A., Sanchez E.L., Camarda R., Lagunoff M. (2015). Dengue Virus Induces and Requires Glycolysis for Optimal Replication. J. Virol..

[13] Yogev O., Lagos D., Enver T., Boshoff C. (2014). Kaposi’s sarcoma herpesvirus microRNAs induce metabolic transformation of infected cells. PLoS Pathog..

[14] Diamond D.L. (2010). Temporal Proteome and Lipidome Profiles Reveal Hepatitis C Virus-Associated Reprogramming of Hepatocellular Metabolism and Bioenergetics. PLoS Pathog..

[15] Fontaine K.A., Camarda R., Lagunoff M. (2014). Vaccinia Virus Requires Glutamine but Not Glucose for Efficient Replication. Journal of Virology.

[16] Hegedus A., Kavanagh Williamson M., Huthoff H. (2014). HIV-1 pathogenicity and virion production are dependent on the metabolic phenotype of activated CD4+ T cells. Retrovirology.

[17] Ritter J.B., Wahl A.S., Freund S., Genzel Y., Reichl U. (2010). Metabolic effects of influenza virus infection in cultured animal cells: Intra- and extracellular metabolite profiling. BMC Syst. Biol..

[18] Vastag L., Koyuncu E., Grady S.L., Shenk T.E., Rabinowitz J.D. (2011). Divergent effects of human cytomegalovirus and herpes simplex virus-1 on cellular metabolism. PLoS Pathog..

[19] Vander Heiden M.G., Cantley L.C., Thompson C.B. (2009). Understanding the Warburg effect: The metabolic requirements of cell proliferation. Science.

[20] Warburg O. (1956). On the origin of cancer cells. Science.

[21] Goodwin C.M., Xu S., Munger J. (2015). Stealing the Keys to the Kitchen: Viral Manipulation of the Host Cell Metabolic Network. Trends Microbiol..

[22] Sanchez E.L., Lagunoff M. (2015). Viral activation of cellular metabolism. Virology.

[23] Fritsch S.D., Weichhart T. (2016). Effects of Interferons and Viruses on Metabolism. Front. Immunol..

[24] Sanchez E.L., Pulliam T.H., Dimaio T.A., Thalhofer A.B., Delgado T., Lagunoff M. (2017). Glycolysis, Glutaminolysis, and Fatty Acid Synthesis Are Required for Distinct Stages of Kaposi’s Sarcoma-Associated Herpesvirus Lytic Replication. J. Virol..

[25] Isaacs A., Lindenmann J. (1987). Virus interference. I. The interferon. By A. Isaacs and J. Lindenmann, 1957. J. Interferon Res..

[26] Schneider W.M., Chevillotte M.D., Rice C.M. (2014). Interferon-Stimulated Genes: A Complex Web of Host Defenses. Annu. Rev. Immunol..

[27] Li M.M., MacDonald M.R., Rice C.M. (2015). To translate, or not to translate: Viral and host mRNA regulation by interferon-stimulated genes. Trends Cell Biol..

[28] Schoggins J.W., MacDuff D.A., Imanaka N., Gainey M.D., Shrestha B., Eitson J.L., Mar K.B., Richardson R.B., Ratushny A.V., Litvak V. (2014). Pan-viral specificity of IFN-induced genes reveals new roles for cGAS in innate immunity. Nature.

[29] Schoggins J.W., Wilson S.J., Panis M., Murphy M.Y., Jones C.T., Bieniasz P., Rice C.M. (2011). A diverse range of gene products are effectors of the type I interferon antiviral response. Nature.

[30] Kane M., Zang T.M., Rihn S.J., Zhang F., Kueck T., Alim M., Schoggins J., Rice C.M., Wilson S.J., Bieniasz P.D. (2016). Identification of Interferon-Stimulated Genes with Antiretroviral Activity. Cell Host Microbe.

[31] Liu S.Y., Sanchez D.J., Aliyari R., Lu S., Cheng G. (2012). Systematic identification of type I and type II interferon-induced antiviral factors. Proc. Natl. Acad. Sci. USA.

[32] Lu J., Pan Q., Rong L., He W., Liu S.L., Liang C. (2011). The IFITM proteins inhibit HIV-1 infection. J. Virol..

[33] Yoneyama M., Fujita T. (2007). RIG-I family RNA helicases: Cytoplasmic sensor for antiviral innate immunity. Cytokine Growth Factor Rev..

[34] Ahlers L.R.H., Goodman A.G. (2016). Nucleic acid sensing and innate immunity: Signaling pathways controlling viral pathogenesis and autoimmunity. Curr. Clin. Microbiol. Rep..

[35] Heaton N.S., Randall G. (2011). Multifaceted roles for lipids in viral infection. Trends Microbiol..

[36] Jackel-Cram C., Qiao L., Xiang Z., Brownlie R., Zhou Y., Babiuk L., Liu Q. (2010). Hepatitis C virus genotype-3a core protein enhances sterol regulatory element-binding protein-1 activity through the phosphoinositide 3-kinase-Akt-2 pathway. J. Gen. Virol..

[37] Blanc M., Hsieh W.Y., Robertson K.A., Watterson S., Shui G., Lacaze P., Khondoker M., Dickinson P., Sing G., Rodríguez-Martín S. (2011). Host Defense against Viral Infection Involves Interferon Mediated Down-Regulation of Sterol Biosynthesis. PLoS Biol..

[38] Liu S.Y., Aliyari R., Chikere K., Li G., Marsden M.D., Smith J.K., Pernet O., Guo H., Nusbaum R., Zack J.A. (2013). Interferon-inducible cholesterol-25-hydroxylase broadly inhibits viral entry by production of 25-hydroxycholesterol. Immunity.

[39] Brown M.S., Goldstein J.L. (1997). The SREBP pathway: Regulation of cholesterol metabolism by proteolysis of a membrane-bound transcription factor. Cell.

[40] Singaravelu R., O’Hara S., Jones D.M., Chen R., Taylor N.G., Srinivasan P., Quan C., Roy D.G., Steenbergen R.H., Kumar A. (2015). MicroRNAs regulate the immunometabolic response to viral infection in the liver. Nat. Chem. Biol..

[41] Moog C., Aubertin A.M., Kirn A., Luu B. (1998). Oxysterols, but not cholesterol, inhibit human immunodeficiency virus replication in vitro. Antivir. Chem. Chemother..

[42] Anggakusuma, Romero-Brey I., Berger C., Colpitts C.C., Boldanova T., Engelmann M., Todt D., Perin P.M., Behrendt P., Vondran F.W., Xu S. (2015). Interferon-inducible cholesterol-25-hydroxylase restricts hepatitis C virus replication through blockage of membranous web formation. Hepatology.

[43] Tani H., Shimojima M., Fukushi S., Yoshikawa T., Fukuma A., Taniguchi S., Morikawa S., Saijo M. (2016). Characterization of Glycoprotein-Mediated Entry of Severe Fever with Thrombocytopenia Syndrome Virus. J. Virol..

[44] Li C., Deng Y.Q., Wang S., Ma F., Aliyari R., Huang X.Y., Zhang N.N., Watanabe M., Dong H.L., Liu P. (2017). 25-Hydroxycholesterol Protects Host against Zika Virus Infection and Its Associated Microcephaly in a Mouse Model. Immunity.

[45] Wang J., Zeng L., Zhang L., Guo Z.Z., Lu S.F., Ming S.L., Li G.L., Wan B., Tian K.G., Yang G.Y. (2017). Cholesterol 25-hydroxylase acts as a host restriction factor on pseudorabies virus replication. J. Gen. Virol..

[46] Lembo D., Cagno V., Civra A., Poli G. (2016). Oxysterols: An emerging class of broad spectrum antiviral effectors. Mol. Aspects Med..

[47] Civra A., Cagno V., Donalisio M., Biasi F., Leonarduzzi G., Poli G., Lembo D. (2014). Inhibition of pathogenic non-enveloped viruses by 25-hydroxycholesterol and 27-hydroxycholesterol. Sci. Rep..

[48] Song Z., Zhang Q., Liu X., Bai J., Zhao Y., Wang X., Jiang P. (2017). Cholesterol 25-hydroxylase is an interferon-inducible factor that protects against porcine reproductive and respiratory syndrome virus infection. Vet. Microbiol..

[49] Pereiro P., Forn-Cuni G., Dios S., Coll J., Figueras A., Novoa B. (2017). Interferon-independent antiviral activity of 25-hydroxycholesterol in a teleost fish. Antivir. Res..

[50] Vigerust D.J., Shepherd V.L. (2007). Virus glycosylation: Role in virulence and immune interactions. Trends Microbiol..

[51] Shrivastava-Ranjan P., Bergeron E., Chakrabarti A.K., Albarino C.G., Flint M., Nichol S.T., Spiropoulou C.F. (2016). 25-Hydroxycholesterol Inhibition of Lassa Virus Infection through Aberrant GP1 Glycosylation. mBio.

[52] Burri D.J., da Palma J.R., Kunz S., Pasquato A. (2012). Envelope glycoprotein of arenaviruses. Viruses.

[53] Cagno V., Civra A., Rossin D., Calfapietra S., Caccia C., Leoni V., Dorma N., Biasi F., Poli G., Lembo D. (2017). Inhibition of herpes simplex-1 virus replication by 25-hydroxycholesterol and 27-hydroxycholesterol. Redox Biol..

[54] Arita M., Kojima H., Nagano T., Okabe T., Wakita T., Shimizu H. (2013). Oxysterol-binding protein family I is the target of minor enviroxime-like compounds. J. Virol..

[55] Olkkonen V.M., Li S. (2013). Oxysterol-binding proteins: Sterol and phosphoinositide sensors coordinating transport, signaling and metabolism. Prog. Lipid Res..

[56] Arita M. (2014). Phosphatidylinositol-4 kinase III beta and oxysterol-binding protein accumulate unesterified cholesterol on poliovirus-induced membrane structure. Microbiol. Immunol..

[57] Roulin P.S., Lotzerich M., Torta F., Tanner L.B., van Kuppeveld F.J., Wenk M.R., Greber U.F. (2014). Rhinovirus uses a phosphatidylinositol 4-phosphate/cholesterol counter-current for the formation of replication compartments at the ER-Golgi interface. Cell Host Microbe.

[58] Funk J.L., Feingold K.R., Moser A.H., Grunfeld C. (1993). Lipopolysaccharide stimulation of RAW 264.7 macrophages induces lipid accumulation and foam cell formation. Atherosclerosis.

[59] Huang Y.L., Morales-Rosado J., Ray J., Myers T.G., Kho T., Lu M., Munford R.S. (2014). Toll-like receptor agonists promote prolonged triglyceride storage in macrophages. J. Biol. Chem..

[60] Dushkin M.I., Kovshik G.G. (2013). Effect of toll-like receptor agonists on the formation of macrophage/foam cells upon acute peritonitis in mice. Bull. Exp. Biol. Med..

[61] York Autumn G., Williams Kevin J., Argus Joseph P., Zhou Quan D., Brar G., Vergnes L., Gray Elizabeth E., Zhen A., Wu Nicholas C., Yamada Douglas H. (2015). Limiting Cholesterol Biosynthetic Flux Spontaneously Engages Type I IFN Signaling. Cell.

[62] Li M.M., MacDonald M.R. (2016). Polyamines: Small Molecules with a Big Role in Promoting Virus Infection. Cell Host Microbe.

[63] Mounce B.C., Olsen M.E., Vignuzzi M., Connor J.H. (2017). Polyamines and Their Role in Virus Infection. Microbiol. Mol. Biol. Rev..

[64] Pegg A.E. (2009). Mammalian polyamine metabolism and function. IUBMB Life.

[65] Schipper R.G., Penning L.C., Verhofstad A.A. (2000). Involvement of polyamines in apoptosis. Facts and controversies: Effectors or protectors?. Semin. Cancer Biol..

[66] Ha H.C., Sirisoma N.S., Kuppusamy P., Zweier J.L., Woster P.M., Casero R.A. (1998). The natural polyamine spermine functions directly as a free radical scavenger. Proc. Natl. Acad. Sci. USA.

[67] Childs A.C., Mehta D.J., Gerner E.W. (2003). Polyamine-dependent gene expression. Cell. Mol. Life Sci..

[68] Mounce B.C., Poirier E.Z., Passoni G., Simon-Loriere E., Cesaro T., Prot M., Stapleford K.A., Moratorio G., Sakuntabhai A., Levraud J.P. (2016). Interferon-Induced Spermidine-Spermine Acetyltransferase and Polyamine Depletion Restrict Zika and Chikungunya Viruses. Cell Host Microbe.

[69] Rusinova I., Forster S., Yu S., Kannan A., Masse M., Cumming H., Chapman R., Hertzog P.J. (2013). Interferome v2.0: An updated database of annotated interferon-regulated genes. Nucleic Acids Res..

[70] Lightfoot H.L., Hall J. (2014). Endogenous polyamine function—The RNA perspective. Nucleic Acids Res..

[71] Igarashi K., Kashiwagi K. (2015). Modulation of protein synthesis by polyamines. IUBMB Life.

[72] Gibson W., Roizman B. (1971). Compartmentalization of spermine and spermidine in the herpes simplex virion. Proc. Natl. Acad. Sci. USA.

[73] Tyms A.S., Williamson J.D. (1982). Inhibitors of polyamine biosynthesis block human cytomegalovirus replication. Nature.

[74] Lanzer W., Holowczak J.A. (1975). Polyamines in vaccinia virions and polypeptides released from viral cores by acid extraction. J. Virol..

[75] Park M.H., Cooper H.L., Folk J.E. (1981). Identification of hypusine, an unusual amino acid, in a protein from human lymphocytes and of spermidine as its biosynthetic precursor. Proc. Natl. Acad. Sci. USA.

[76] Park M.H. (2006). The post-translational synthesis of a polyamine-derived amino acid, hypusine, in the eukaryotic translation initiation factor 5A (eIF5A). J. Biochem..

[77] Olsen M.E., Filone C.M., Rozelle D., Mire C.E., Agans K.N., Hensley L., Connor J.H. (2016). Polyamines and Hypusination Are Required for Ebolavirus Gene Expression and Replication. mBio.

[78] Baumann S., Sander A., Gurnon J.R., Yanai-Balser G., VanEtten J.L., Piotrowski M. (2007). Chlorella viruses contain genes encoding a complete polyamine biosynthetic pathway. Virology.

[79] Smirnova O.A., Keinanen T.A., Ivanova O.N., Hyvonen M.T., Khomutov A.R., Kochetkov S.N., Bartosch B., Ivanov A.V. (2017). Hepatitis C virus alters metabolism of biogenic polyamines by affecting expression of key enzymes of their metabolism. Biochem. Biophys. Res. Commun..

[80] Evageliou N.F., Haber M., Vu A., Laetsch T.W., Murray J., Gamble L.D., Cheng N.C., Liu K., Reese M., Corrigan K.A. (2016). Polyamine Antagonist Therapies Inhibit Neuroblastoma Initiation and Progression. Clin. Cancer Res..

[81] Bassiri H., Benavides A., Haber M., Gilmour S.K., Norris M.D., Hogarty M.D. (2015). Translational development of difluoromethylornithine (DFMO) for the treatment of neuroblastoma. Transl. Pediatr..

[82] Mounce B.C., Cesaro T., Moratorio G., Hooikaas P.J., Yakovleva A., Werneke S.W., Smith E.C., Poirier E.Z., Simon-Loriere E., Prot M. (2016). Inhibition of Polyamine Biosynthesis Is a Broad-Spectrum Strategy against RNA Viruses. J. Virol..

[83] Creaven P.J., Pendyala L., Petrelli N.J. (1993). Evaluation of alpha-difluoromethylornithine as a potential chemopreventive agent: Tolerance to daily oral administration in humans. Cancer Epidemiol. Biomark. Prev..

[84] Sainio E.L., Pulkki K., Young S.N. (1996). L-Tryptophan: Biochemical, nutritional and pharmacological aspects. Amino Acids.

[85] Moffett J.R., Namboodiri M.A. (2003). Tryptophan and the immune response. Immunol. Cell Biol..

[86] Leklem J.E. (1971). Quantitative aspects of tryptophan metabolism in humans and other species: A review. Am. J. Clin. Nutr..

[87] King N.J., Thomas S.R. (2007). Molecules in focus: Indoleamine 2,3-dioxygenase. Int. J. Biochem. Cell Biol..

[88] Fukunaga M., Yamamoto Y., Kawasoe M., Arioka Y., Murakami Y., Hoshi M., Saito K. (2012). Studies on tissue and cellular distribution of indoleamine 2,3-dioxygenase 2: The absence of IDO1 upregulates IDO2 expression in the epididymis. J. Histochem. Cytochem..

[89] Robinson C.M., Shirey K.A., Carlin J.M. (2003). Synergistic transcriptional activation of indoleamine dioxygenase by IFN-gamma and tumor necrosis factor-alpha. J. Interferon Cytokine Res..

[90] Manlapat A.K., Kahler D.J., Chandler P.R., Munn D.H., Mellor A.L. (2007). Cell-autonomous control of interferon type I expression by indoleamine 2,3-dioxygenase in regulatory CD19+ dendritic cells. Eur. J. Immunol..

[91] Fox J.M., Crabtree J.M., Sage L.K., Tompkins S.M., Tripp R.A. (2015). Interferon Lambda Upregulates IDO1 Expression in Respiratory Epithelial Cells After Influenza Virus Infection. J. Interferon Cytokine Res..

[92] Adams O., Besken K., Oberdorfer C., MacKenzie C.R., Russing D., Daubener W. (2004). Inhibition of human herpes simplex virus type 2 by interferon gamma and tumor necrosis factor alpha is mediated by indoleamine 2,3-dioxygenase. Microbes Infect..

[93] Iwamoto N., Ito H., Ando K., Ishikawa T., Hara A., Taguchi A., Saito K., Takemura M., Imawari M., Moriwaki H. (2009). Upregulation of indoleamine 2,3-dioxygenase in hepatocyte during acute hepatitis caused by hepatitis B virus-specific cytotoxic T lymphocytes in vivo. Liver Int..

[94] Larrea E., Riezu-Boj J.I., Gil-Guerrero L., Casares N., Aldabe R., Sarobe P., Civeira M.P., Heeney J.L., Rollier C., Verstrepen B. (2007). Upregulation of indoleamine 2,3-dioxygenase in hepatitis C virus infection. J. Virol..

[95] Terajima M., Leporati A.M. (2005). Role of Indoleamine 2,3-Dioxygenase in Antiviral Activity of Interferon-gamma Against Vaccinia Virus. Viral Immunol..

[96] Rabbani M.A., Ribaudo M., Guo J.T., Barik S. (2016). Identification of Interferon-Stimulated Gene Proteins That Inhibit Human Parainfluenza Virus Type 3. J. Virol..

[97] Schmidt S.V., Schultze J.L. (2014). New Insights into IDO Biology in Bacterial and Viral Infections. Front. Immunol..

[98] Gaelings L., Soderholm S., Bugai A., Fu Y., Nandania J., Schepens B., Lorey M.B., Tynell J., Vande Ginste L., Le Goffic R. (2017). Regulation of kynurenine biosynthesis during influenza virus infection. FEBS J..

[99] Huang L., Li L., Klonowski K.D., Tompkins S.M., Tripp R.A., Mellor A.L. (2013). Induction and role of indoleamine 2,3 dioxygenase in mouse models of influenza a virus infection. PLoS ONE.

[100] Panasiuk A., Prokopowicz D., Zak J., Matowicka-Karna J., Osada J., Wysocka J. (2001). Activation of blood platelets in chronic hepatitis and liver cirrhosis P-selectin expression on blood platelets and secretory activity of beta-thromboglobulin and platelet factor-4. Hepatogastroenterology.

[101] Iannacone M., Sitia G., Isogawa M., Whitmire J.K., Marchese P., Chisari F.V., Ruggeri Z.M., Guidotti L.G. (2008). Platelets prevent IFN-alpha/beta-induced lethal hemorrhage promoting CTL-dependent clearance of lymphocytic choriomeningitis virus. Proc. Natl. Acad. Sci. USA.

[102] Lang P.A., Contaldo C., Georgiev P., El-Badry A.M., Recher M., Kurrer M., Cervantes-Barragan L., Ludewig B., Calzascia T., Bolinger B. (2008). Aggravation of viral hepatitis by platelet-derived serotonin. Nat. Med..

[103] Boasso A., Herbeuval J.P., Hardy A.W., Anderson S.A., Dolan M.J., Fuchs D., Shearer G.M. (2007). HIV inhibits CD4+ T-cell proliferation by inducing indoleamine 2,3-dioxygenase in plasmacytoid dendritic cells. Blood.

[104] Boasso A., Hardy A.W., Anderson S.A., Dolan M.J., Shearer G.M. (2008). HIV-induced type I interferon and tryptophan catabolism drive T cell dysfunction despite phenotypic activation. PLoS ONE.

[105] Drewes J.L., Croteau J.D., Shirk E.N., Engle E.L., Zink M.C., Graham D.R. (2016). Distinct Patterns of Tryptophan Maintenance in Tissues during Kynurenine Pathway Activation in Simian Immunodeficiency Virus-Infected Macaques. Front. Immunol..

[106] Mbongue J.C., Nicholas D.A., Torrez T.W., Kim N.-S., Firek A.F., Langridge W.H.R. (2015). The Role of Indoleamine 2, 3-Dioxygenase in Immune Suppression and Autoimmunity. Vaccines.

[107] Uyttenhove C., Pilotte L., Theate I., Stroobant V., Colau D., Parmentier N., Boon T., Van den Eynde B.J. (2003). Evidence for a tumoral immune resistance mechanism based on tryptophan degradation by indoleamine 2,3-dioxygenase. Nat. Med..

[108] Yoshida R., Urade Y., Tokuda M., Hayaishi O. (1979). Induction of indoleamine 2,3-dioxygenase in mouse lung during virus infection. Proc. Natl. Acad. Sci. USA.

[109] Guillonneau C., Mintern J.D., Hubert F.X., Hurt A.C., Besra G.S., Porcelli S., Barr I.G., Doherty P.C., Godfrey D.I., Turner S.J. (2009). Combined NKT cell activation and influenza virus vaccination boosts memory CTL generation and protective immunity. Proc. Natl. Acad. Sci. USA.

[110] Fallarini S., Paoletti T., Panza L., Lombardi G. (2008). Alpha-galactosylceramide modulates the induction of indoleamine 2,3-dioxygenase in antigen presenting cells. Biochem. Pharmacol..

[111] Fox J.M., Sage L.K., Huang L., Barber J., Klonowski K.D., Mellor A.L., Tompkins S.M., Tripp R.A. (2013). Inhibition of indoleamine 2,3-dioxygenase enhances the T-cell response to influenza virus infection. J. Gen. Virol..

[112] Fox J.M., Sage L.K., Poore S., Johnson S., Tompkins S.M., Tripp R.A. (2014). Drug analog inhibition of indoleamine 2,3-dioxygenase (IDO) activity modifies pattern recognition receptor expression and proinflammatory cytokine responses early during influenza virus infection. J. Leukoc. Biol..

[113] Laguette N., Sobhian B., Casartelli N., Ringeard M., Chable-Bessia C., Segeral E. (2011). SAMHD1 is the dendritic- and myeloid-cell-specific HIV-1 restriction factor counteracted by Vpx. Nature.

[114] Hrecka K., Hao C., Gierszewska M., Swanson S.K., Kesik-Brodacka M., Srivastava S. (2011). Vpx relieves inhibition of HIV-1 infection of macrophages mediated by the SAMHD1 protein. Nature.

[115] Gramberg T., Kahle T., Bloch N., Wittmann S., Müllers E., Daddacha W., Hofmann H., Kim B., Lindemann D., Landau N.R. (2013). Restriction of diverse retroviruses by SAMHD1. Retrovirology.

[116] Sze A., Belgnaoui S.M., Olagnier D., Lin R., Hiscott J., van Grevenynghe J. (2013). Host restriction factor SAMHD1 limits human T cell leukemia virus type 1 infection of monocytes via STING-mediated apoptosis. Cell Host Microbe.

[117] Kim E.T., White T.E., Brandariz-Nunez A., Diaz-Griffero F., Weitzman M.D. (2013). SAMHD1 restricts herpes simplex virus 1 in macrophages by limiting DNA replication. J. Virol..

[118] Chen Z., Zhu M., Pan X., Zhu Y., Yan H., Jiang T., Shen Y., Dong X., Zheng N., Lu J. (2014). Inhibition of Hepatitis B virus replication by SAMHD1. Biochem. Biophys. Res. Commun..

[119] Rice G.I., Bond J., Asipu A., Brunette R.L., Manfield I.W., Carr I.M., Fuller J.C., Jackson R.M., Lamb T., Briggs T.A. (2009). Mutations involved in Aicardi-Goutieres syndrome implicate SAMHD1 as regulator of the innate immune response. Nat. Genet..

[120] Goutieres F., Aicardi J., Barth P.G., Lebon P. (1998). Aicardi-Goutieres syndrome: An update and results of interferon-alpha studies. Ann. Neurol..

[121] Li N., Zhang W., Cao X. (2000). Identification of human homologue of mouse IFN-gamma induced protein from human dendritic cells. Immunol. Lett..

[122] Pauls E., Jimenez E., Ruiz A., Permanyer M., Ballana E., Costa H., Nascimiento R., Parkhouse R.M., Pena R., Riveiro-Munoz E. (2013). Restriction of HIV-1 replication in primary macrophages by IL-12 and IL-18 through the upregulation of SAMHD1. J. Immunol..

[123] Riess M., Fuchs N.V., Idica A., Hamdorf M., Flory E., Pedersen I.M., Konig R. (2017). Interferons Induce Expression of SAMHD1 in Monocytes through Down-regulation of miR-181a and miR-30a. J. Biol. Chem..

[124] Jin C., Peng X., Liu F., Cheng L., Xie T., Lu X., Wu H., Wu N. (2016). Interferon-induced sterile alpha motif and histidine/aspartic acid domain-containing protein 1 expression in astrocytes and microglia is mediated by microRNA-181a. Aids.

[125] St Gelais C., de Silva S., Amie S.M., Coleman C.M., Hoy H., Hollenbaugh J.A., Kim B., Wu L. (2012). SAMHD1 restricts HIV-1 infection in dendritic cells (DCs) by dNTP depletion, but its expression in DCs and primary CD4(+) T-lymphocytes cannot be upregulated by interferons. Retrovirology.

[126] Goujon C., Schaller T., Galao R.P., Amie S.M., Kim B., Olivieri K., Neil S.J., Malim M.H. (2013). Evidence for IFNalpha-induced, SAMHD1-independent inhibitors of early HIV-1 infection. Retrovirology.

[127] Goldstone D.C., Ennis-Adeniran V., Hedden J.J., Groom H.C., Rice G.I., Christodoulou E., Walker P.A., Kelly G., Haire L.F., Yap M.W. (2011). HIV-1 restriction factor SAMHD1 is a deoxynucleoside triphosphate triphosphohydrolase. Nature.

[128] Lahouassa H., Daddacha W., Hofmann H., Ayinde D., Logue E.C., Dragin L., Bloch N., Maudet C., Bertrand M., Gramberg T. (2012). SAMHD1 restricts the replication of human immunodeficiency virus type 1 by depleting the intracellular pool of deoxynucleoside triphosphates. Nat. Immunol..

[129] Ryoo J., Choi J., Oh C., Kim S., Seo M., Kim S.Y., Seo D., Kim J., White T.E., Brandariz-Nunez A. (2014). The ribonuclease activity of SAMHD1 is required for HIV-1 restriction. Nat. Med..

[130] Choi J., Ryoo J., Oh C., Hwang S., Ahn K. (2015). SAMHD1 specifically restricts retroviruses through its RNase activity. Retrovirology.

[131] Ryoo J., Hwang S.-Y., Choi J., Oh C., Ahn K. (2016). Reply to SAMHD1-mediated HIV-1 restriction in cells does not involve ribonuclease activity. Nat. Med..

[132] Antonucci J.M., St Gelais C., de Silva S., Yount J.S., Tang C., Ji X., Shepard C., Xiong Y., Kim B., Wu L. (2016). SAMHD1-mediated HIV-1 restriction in cells does not involve ribonuclease activity. Nat. Med..

[133] Wu L. (2013). SAMHD1 knockout mice: Modeling retrovirus restriction in vivo. Retrovirology.

[134] Behrendt R., Schumann T., Gerbaulet A., Nguyen L.A., Schubert N., Alexopoulou D., Berka U., Lienenklaus S., Peschke K., Gibbert K. (2013). Mouse SAMHD1 has antiretroviral activity and suppresses a spontaneous cell-intrinsic antiviral response. Cell Rep..

[135] Yan J., Hao C., DeLucia M., Swanson S., Florens L., Washburn M.P., Ahn J., Skowronski J. (2015). CyclinA2-Cyclin-dependent Kinase Regulates SAMHD1 Protein Phosphohydrolase Domain. J. Biol. Chem..

[136] White T.E., Brandariz-Nunez A., Valle-Casuso J.C., Amie S., Nguyen L.A., Kim B. (2013). The retroviral restriction ability of SAMHD1, but not its deoxynucleotide triphosphohydrolase activity, is regulated by phosphorylation. Cell Host Microbe.

[137] Rocha-Perugini V., Suarez H., Alvarez S., Lopez-Martin S., Lenzi G.M., Vences-Catalan F., Levy S., Kim B., Munoz-Fernandez M.A., Sanchez-Madrid F. (2017). CD81 association with SAMHD1 enhances HIV-1 reverse transcription by increasing dNTP levels. Nat. Microbiol..

[138] Pan X., Baldauf H.M., Keppler O.T., Fackler O.T. (2013). Restrictions to HIV-1 replication in resting CD4(+) T lymphocytes. Cell Res..

[139] Monroe K.M., Yang Z., Johnson J.R., Geng X., Doitsh G., Krogan N.J., Greene W.C. (2014). IFI16 DNA sensor is required for death of lymphoid CD4 T cells abortively infected with HIV. Science.

[140] World Health Organization (2017). World Health Statistics 2017: Monitoring Health for the SDGs, Sustainable Development Goals.

[141] Li T.C., Chan M.C., Lee N. (2015). Clinical Implications of Antiviral Resistance in Influenza. Viruses.

[142] Nguyen H.T., Fry A.M., Gubareva L.V. (2012). Neuraminidase inhibitor resistance in influenza viruses and laboratory testing methods. Antivir. Ther..

[143] Thorlund K., Awad T., Boivin G., Thabane L. (2011). Systematic review of influenza resistance to the neuraminidase inhibitors. BMC Infect. Dis..

[144] Reece P.A. (2007). Neuraminidase inhibitor resistance in influenza viruses. J. Med. Virol..

[145] Smallwood H.S., Duan S., Morfouace M., Rezinciuc S., Shulkin B.L., Shelat A., Zink E.E., Milasta S., Bajracharya R., Oluwaseum A.J. (2017). Targeting Metabolic Reprogramming by Influenza Infection for Therapeutic Intervention. Cell Rep..

[146] Bendell J.C., Kurkjian C., Infante J.R., Bauer T.M., Burris H.A., Greco F.A., Shih K.C., Thompson D.S., Lane C.M., Finney L.H. (2015). A phase 1 study of the sachet formulation of the oral dual PI3K/mTOR inhibitor BEZ235 given twice daily (BID) in patients with advanced solid tumors. Investig. New Drugs.

[147] Polivka J., Janku F. (2014). Molecular targets for cancer therapy in the PI3K/AKT/mTOR pathway. Pharmacol. Ther..

[148] Levy G., Habib N., Guzzardi M.A., Kitsberg D., Bomze D., Ezra E., Uygun B.E., Uygun K., Trippler M., Schlaak J.F. (2016). Nuclear receptors control pro-viral and antiviral metabolic responses to hepatitis C virus infection. Nat. Chem. Biol..

[149] Hayhurst G.P., Lee Y.H., Lambert G., Ward J.M., Gonzalez F.J. (2001). Hepatocyte nuclear factor 4alpha (nuclear receptor 2A1) is essential for maintenance of hepatic gene expression and lipid homeostasis. Mol. Cell. Biol..

[150] Bonzo J.A., Ferry C.H., Matsubara T., Kim J.H., Gonzalez F.J. (2012). Suppression of hepatocyte proliferation by hepatocyte nuclear factor 4alpha in adult mice. J. Biol. Chem..

[151] Holloway M.G., Miles G.D., Dombkowski A.A., Waxman D.J. (2008). Liver-specific hepatocyte nuclear factor-4alpha deficiency: Greater impact on gene expression in male than in female mouse liver. Mol. Endocrinol..

[152] Long Y.C., Zierath J.R. (2006). AMP-activated protein kinase signaling in metabolic regulation. J. Clin. Investig..

[153] Hardie D.G., Pan D.A. (2002). Regulation of fatty acid synthesis and oxidation by the AMP-activated protein kinase. Biochem. Soc. Trans..

[154] Moser T.S., Schieffer D., Cherry S. (2012). AMP-Activated Kinase Restricts Rift Valley Fever Virus Infection by Inhibiting Fatty Acid Synthesis. PLoS Pathog..

[155] Cheng F., He M., Jung J.U., Lu C., Gao S.J. (2016). Suppression of Kaposi’s Sarcoma-Associated Herpesvirus Infection and Replication by 5′-AMP-Activated Protein Kinase. J. Virol..

[156] Pold R., Jensen L.S., Jessen N., Buhl E.S., Schmitz O., Flyvbjerg A., Fujii N., Goodyear L.J., Gotfredsen C.F., Brand C.L. (2005). Long-term AICAR administration and exercise prevents diabetes in ZDF rats. Diabetes.

[157] Idrovo J.P., Yang W.L., Jacob A., Aziz M., Nicastro J., Coppa G.F., Wang P. (2015). AICAR attenuates organ injury and inflammatory response after intestinal ischemia and reperfusion. Mol. Med..

[158] Mounce B.C., Cesaro T., Vlajnić L., Vidiņa A., Vallet T., Weger-Lucarelli J., Passoni G., Stapleford K.A., Levraud J.P., Vignuzzi M. (2017). Chikungunya Virus Overcomes Polyamine Depletion by Mutation of nsP1 and the Opal Stop Codon To Confer Enhanced Replication and Fitness. J. Virol..

